# Current Status and Priorities of Valved Conduits for Right Ventricle-to-Pulmonary Artery Reconstruction in Japan: A Nationwide Survey

**DOI:** 10.1093/icvts/ivag177

**Published:** 2026-06-24

**Authors:** Shintaro Nemoto, Hayato Konishi, Akiyo Suzuki, Takahiro Katsumata, Masaru Miura, Yusei Hamada, Yasumi Nishiwaki

**Affiliations:** Department of Thoracic and Cardiovascular Surgery, Osaka Medical and Pharmaceutical University, Takatsuki, Osaka 569-8686, Japan; Department of Thoracic and Cardiovascular Surgery, Osaka Medical and Pharmaceutical University, Takatsuki, Osaka 569-8686, Japan; Department of Thoracic and Cardiovascular Surgery, Osaka Medical and Pharmaceutical University, Takatsuki, Osaka 569-8686, Japan; Department of Thoracic and Cardiovascular Surgery, Osaka Medical and Pharmaceutical University, Takatsuki, Osaka 569-8686, Japan; Department of Pediatrics, Tokyo Metropolitan Tama-Nambu Chiiki Hospital, Tama, Tokyo 206-0039, Japan; Research & Development Department, Regenerative Medicine & Implantable Medical Device Division, Teijin Limited, Chiyoda, Tokyo 100-8585, Japan; Research & Development Department, Regenerative Medicine & Implantable Medical Device Division, Teijin Limited, Chiyoda, Tokyo 100-8585, Japan

**Keywords:** congenital heart surgery, right ventricle-to-pulmonary artery reconstruction, expanded polytetrafluoroethylene, valved conduit, nationwide survey, durability

## Abstract

Right ventricle-to-pulmonary artery reconstruction is essential in congenital heart surgery. In Japan, cryopreserved homograft availability is limited, necessitating alternative materials. This study systematically elucidated the status of right ventricle-to-pulmonary artery conduit use and surgeon priorities in Japan. In 2024, a web-based nationwide survey was conducted by the Japanese Society of Paediatric Cardiology and Cardiac Surgery, targeting 126 institutions. We analysed data from 2021 to 2023 regarding primary diagnoses, conduit types, and selection criteria. Responses were obtained from 52 institutions (41.2%), covering 926 cases. Handmade expanded polytetrafluoroethylene valved conduits were most frequently used (80.7%), whereas bovine jugular vein conduits (7.5%) and homografts (1.1%) were rarely employed. “Functional Durability and Performance” was the most prioritized criterion (median score: 4.00), with a median expected durability of 10 years. We concluded that in Japan, where a homograft supply system is not established, handmade expanded polytetrafluoroethylene conduits are the primary choice despite their off-label status. A significant gap exists between current performance of conduits in general and clinical expectations for durability, underscoring the need for technological advancements in conduit design.

## Graphical abstract

**Figure:**
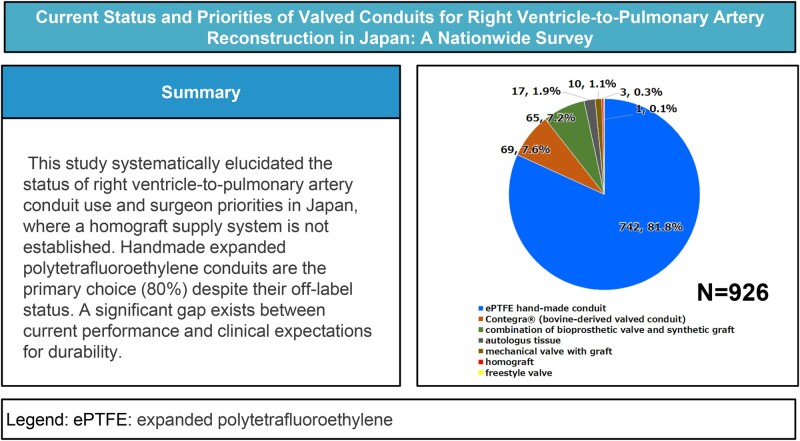

## INTRODUCTION

**Right ventricle-to-pulmonary artery (RV-PA)** reconstruction is a cornerstone of congenital cardiac surgery for conditions such as pulmonary atresia with ventricular septal defect (PA/VSD), tetralogy of Fallot (TOF), and pulmonary position replacement after the Ross procedure. In Europe and the United States, cryopreserved homografts are the established gold standard due to their superior handling, favourable haemodynamics, and proven long-term performance.^1^^,^^2^

In stark contrast, the clinical landscape in Japan is fundamentally different. Homograft availability is extremely limited due to profound donor shortages stemming from cultural and ethical issues surrounding cadaveric donation, alongside constraints in national tissue banking. This unique environment has necessitated the adoption of alternative materials, creating a distinct surgical paradigm that deviates from Western standards. Currently, the bovine jugular vein conduit (Contegra, Medtronic Inc) is the only regulatory-approved RV-PA conduit in Japan; however, its use is often limited by concerns regarding early leaflet dysfunction and progressive calcification.^1^^,^^3^^,^^4^

Consequently, Japanese surgeons have pioneered the use of handmade valved conduits constructed from expanded polytetrafluoroethylene (ePTFE).^5^^,^^6^ While these have gained global popularity as an off-label alternative, their handcrafted nature poses challenges regarding structural uniformity and material-specific degradation.^5^^,^^7^ Currently, there is a critical knowledge gap regarding the nationwide prevalence of these strategies and the performance requirements prioritized by surgeons under such resource-constrained conditions. We conducted a nationwide survey through the Japanese Society of Paediatric Cardiology and Cardiac Surgery to define clinical expectations for next-generation materials in Japan.

## METHODS

### Ethical statement

The study protocol was approved by the clinical research committee of the Japanese Society of Paediatric Cardiology and Cardiac Surgery (JSPCCS) with wavier of informed consent. Because this survey consisted only of the count values provided by each facility described below and responses to the requirements for the conduit, individual patients cannot be identified.

In June and July 2024, the JSPCCS conducted an online questionnaire survey targeting 126 surgical institutions affiliated with the society’s council members.

The survey investigated the clinical use of valved conduits for RV-PA reconstruction over a 3-year period (2021-2023), focusing on: surgical volume, primary diagnoses, initial or replacement, conduit types, and size distribution. Creation of a new pulmonary trunk using autologous tissue and a monocusped transannular patch has been added as an alternative approach without the use of conduits.

Additionally, we assessed prioritized selection criteria and expected durability for conduits in general (**Table S1**). Characteristics were categorized into 6 domains: (1) Usability and Operability, (2) Biocompatibility and Safety, (3) Functional Durability and Performance, (4) Medication Management and Postoperative Care, (5) Anatomical Indications, Limitations, and Size Variations, and (6) Cost-effectiveness and Availability (**Table S2**).

### Statistics

Data were collected using numerical entries, multiple-choice formats, or ranking scales and summarized using descriptive statistics. To ensure data integrity, responses regarding diagnoses, surgical volume, and conduit types were considered valid only when consistent with the total surgical volume reported by each institution.

## RESULTS

### Primary diagnoses and surgical volume

**Figure 1.**
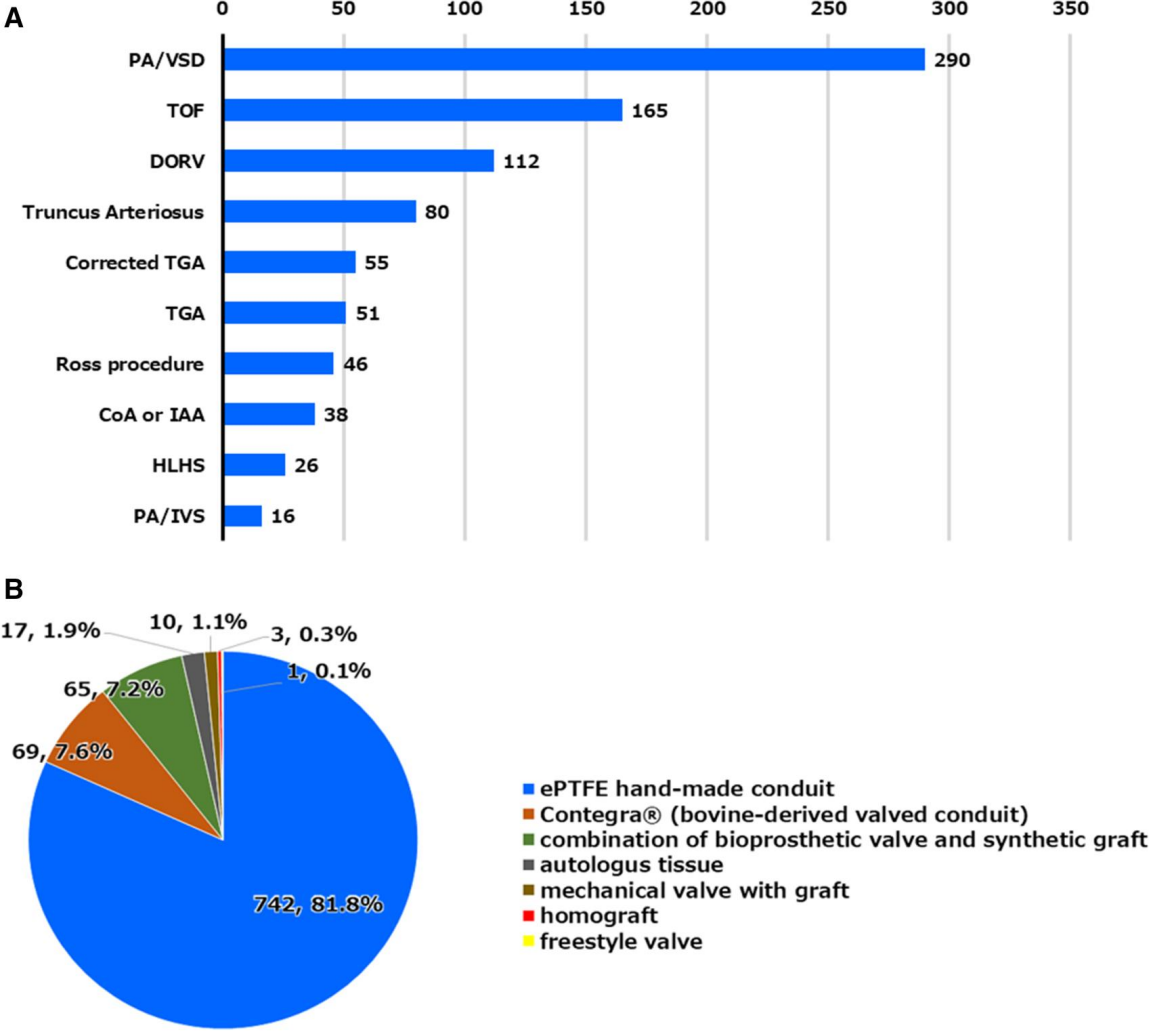
Details of Surgeries Using Valved Conduits: (A) Primary diagnoses and (B) distribution of conduit types. Abbreviations: CoA = coarctation of the aorta; DORV = double outlet right ventricle; ePTFE = expanded polytetrafluoroethylene; HLHS = hypoplastic left heart syndrome; IAA = interrupted aortic arch; PA/IVS = pulmonary atresia with intact ventricular septum; PA/VSD = pulmonary atresia with ventricular septal defect; TGA = transposition of the great arteries; TOF = tetralogy of Fallot.

Responses were obtained from 52 institutions (41.2%), covering 926 RV-PA reconstruction cases. The most common primary diagnoses were PA/VSD (*n* = 290, 31.3%), TOF (*n* = 165, 17.8%), double outlet right ventricle (DORV) (*n* = 112, 12.1%), and cases requiring pulmonary position replacement after the Ross procedure (*n* = 46, 5.0%) (**Figure 1A**). Among 667 cases with valid surgical history data, primary implantation accounted for 60.7% (*n* = 405). Conduit replacements represented 39.3%, comprising first-time (*n* = 222, 33.3%) and subsequent replacements (*n* = 40, 6.0%).

### Distribution of conduit types

Regarding the types of conduits utilized (*n* = 919 valid responses), the handmade ePTFE valved conduit was the most frequently employed (*n* = 742, 80.7%), followed by the bovine jugular vein conduit (*n* = 69, 7.5%) and handmade combinations of synthetic grafts with bioprosthetic valves (*n* = 65, 7.1%) (**Figure 1B**). Despite their prevalence in Western countries, homograft use was documented in only 10 cases (1.1%), restricted to specialized facilities with internal tissue banking.^8^ Notably, autologous tissue repair without a conduit was performed in 17 cases (1.9%). Regarding size distribution, the 16-mm conduit was most frequently utilized, accounting for 64.8% (**Figure S1**).

### Criteria for RV-PA conduit selection

**Figure 2.**
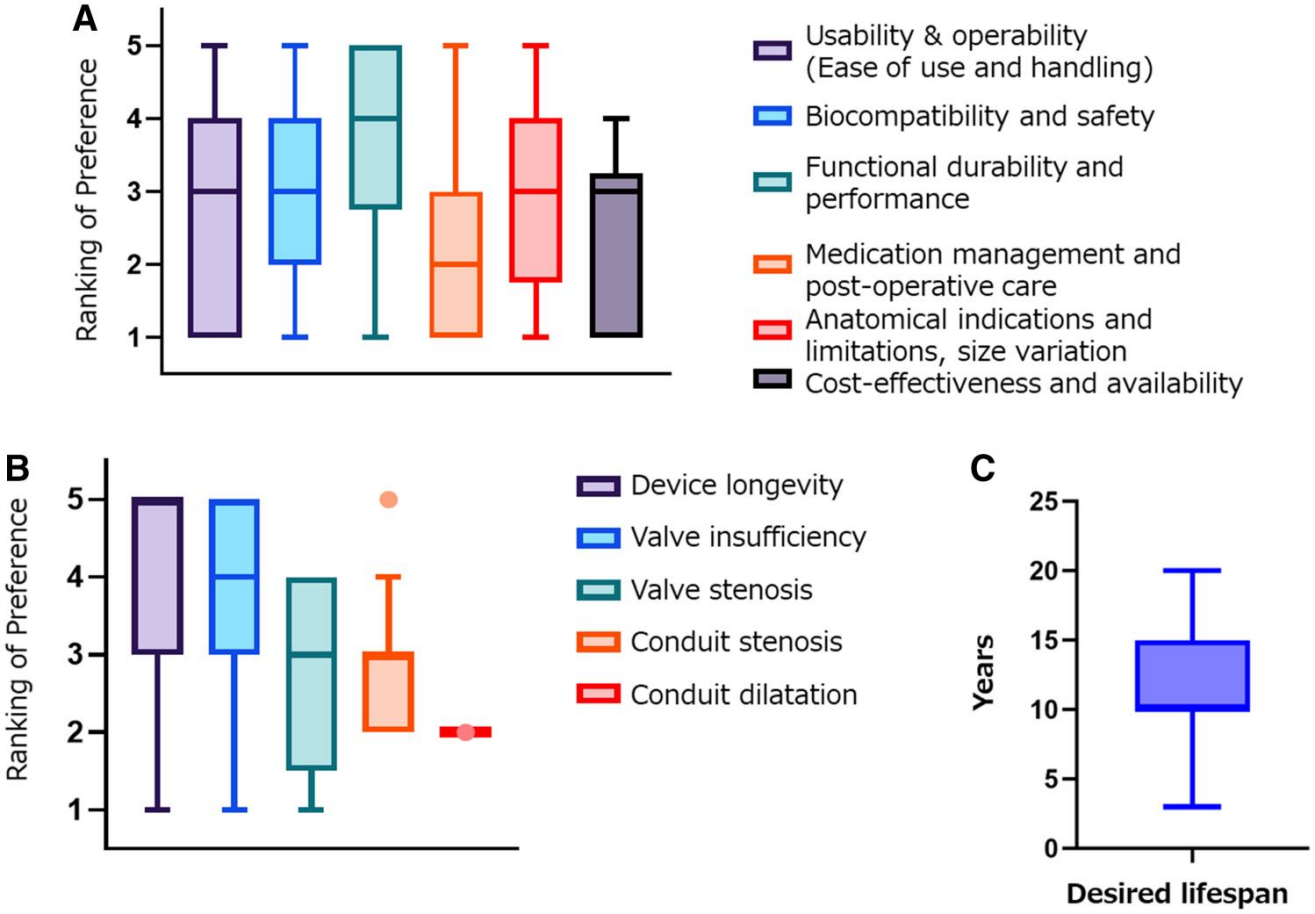
Criteria for Right Ventricle-to-Pulmonary Artery Conduit Selection: (A) Six Major Domains, (B) Sub-Domains of “Functional Durability and Performance,” and (C) Expected Conduit Lifespan.

Evaluation of the 6 predefined selection categories revealed that “Functional Durability and Performance” was the most emphasized domain (median: 4.00; IQR: 2.75-5.00). This was followed by “Biocompatibility and Safety” (3.00; 2.00-4.00), “Anatomical Indications and Limitations” (3.00; 1.75-4.00), and “Usability and Operability” (3.00; 1.00-4.00) (**Figure 2A**). Within the durability category, device longevity was identified as the most critical factor (median: 5.00; IQR: 3.00-5.00), with a median expected durability of 10.0 years (IQR: 10.0-15.0) (**Figure 2B and C**).

## DISCUSSION

This study represents a unique systematic investigation into the nationwide status of valved conduit use for RV-PA reconstruction in Japan, highlighting a distinct surgical paradigm. Two primary findings emerged: first, the vast majority of conduits used are off-label, handmade ePTFE valved conduits; second, functional durability is the paramount priority for surgeons, revealing a significant gap between clinical expectations and current technological reality.

The finding that approximately 80% of conduits were handmade ePTFE represents a profound deviation from the clinical landscape in Europe and the United States, where cryopreserved homografts remain the gold standard. This disparity reflects severe constraints in Japan’s donor availability and the lack of a national tissue banking system. In response, Japanese surgeons have pioneered handmade constructs. While these offer flexible sizing and excellent operability, their handcrafted nature poses challenges regarding structural uniformity. Since this variability may influence predictability of long-term durability, nationwide standardization of handmade successful protocols or products with standardized specifications and guaranteed quality is expected.

Our findings also elucidated that “Functional Durability and Performance” is the most critical domain, with a median expected durability exceeding 10 years. This reflects a global challenge: reducing the cumulative burden of reoperations to improve long-term prognosis. However, existing options—including handmade ePTFE and bovine jugular vein conduits—frequently encounter early leaflet dysfunction or calcification. The 10-year durability identified here serves as a benchmark for future device development. Achieving this longevity will require advanced anti-calcification strategies and optimized leaflet designs specifically tailored to paediatric haemodynamics.

In summary, conduit selection in Japan is heavily dictated by supply constraints, fostering a unique reliance on handmade ePTFE techniques. These results emphasize an urgent demand for next-generation, high-performance conduits that meet the rigorous durability requirements of the surgical community.

### Limitations

This study has several limitations. First, the 41.2% response rate may not fully capture the nationwide situation, potentially introducing bias of conduit selection based on institutional case-volume. However, post-hoc analysis using the median number of conduit implants (11.5 cases/3 years) as a threshold showed no significant difference in the use for ePTFE (81.0% vs 79.4%) vs Contegra (7.9% vs 5.1%) between higher-case (>12, total 783 cases) and lower-case (<11, total 136 cases) institutions. Second, because this survey was cross-sectional, it cannot provide additional longitudinal data regarding the timeframe of dysfunction of each conduits or comparisons thereof. Further prospective studies or utilizing other data sources warrant the results obtained in this study.

## CONCLUSION

This study provides a systematic elucidation of RV-PA reconstruction in Japan. In the absence of a homograft supply system, handmade ePTFE valved conduits—despite their off-label status—have become the primary choice over commercially available products. Although durability is the most prioritized criterion, current options fall short of clinical expectations. These findings indicate a critical need for technological advancements and standardized performance requirements to overcome the limitations of existing RV-PA conduits.

## Supplementary Material

ivag177_Supplementary_Data

## Data Availability

The data related to this article will be shared upon reasonable request to the corresponding author.
