# Supplementary material for: Current Status and Priorities of Valved Conduits for Right Ventricle-to-Pulmonary Artery Reconstruction in Japan: A Nationwide Survey
Source: Interdiscip Cardiovasc Thorac Surg. 2026 Jun 24;41(7):ivag177. doi: 10.1093/icvts/ivag177 (PMC13324388; doi:10.1093/icvts/ivag177)
Supplement: ivag177_Supplementary_Data [file ivag177_supplementary_data.zip › Supplementary Table 1.docx]

**Supplementary Table 1**: Web-based questionnaire

| **No.** | **Questions** |
| --- | --- |
| Q1 | Please indicate the number of surgeries performed using valved conduits in this 3 years.  Please include the number of Rastelli procedures performed using only autologous tissue without conduits. |
| Q2,Q3 | Please indicate　the number of primary diagnoses for the total number of surgeries reported in Q1. |
| Q4 | Please indicate the number of conduit replacement cases of the total number of surgeries reported in Q1 |
| Q5、Q6 | Please indicate the number of each type of valved conduit used in the total number of surgeries reported in Q1. |
| Q7 | Please indicate the size of valved conduit used in the total number of surgeries reported in Q1 |
| Q8、Q9 | Please rank the characteristics in order of importance (1 = highest priority, 5 = fifth priority) when selecting a valved conduit. |
