# Supplementary material for: Current Status and Priorities of Valved Conduits for Right Ventricle-to-Pulmonary Artery Reconstruction in Japan: A Nationwide Survey
Source: Interdiscip Cardiovasc Thorac Surg. 2026 Jun 24;41(7):ivag177. doi: 10.1093/icvts/ivag177 (PMC13324388; doi:10.1093/icvts/ivag177)
Supplement: ivag177_Supplementary_Data [file ivag177_supplementary_data.zip › Supplementary Table 2.docx]

**Supplementary Table 2**: Prioritized selection criteria for a conduit

| **Main Criteria** | **Sub-Criteria** |
| --- | --- |
| Usability & operability | Handling/Needle hole bleeding/Fitting |
| Biocompatibility and safety | Material/Neointimal hyperplasia/Calcification/Adhesion |
| Functional durability and performance | Device longevity/Valve insufficiency/Valve stenosis/  Conduit stenosis/Conduit dilation |
| Medication management and post-operative care | Anticoagulants |
| Anatomical indications and limitations, size variation | Limitation due to blood pressure/Size variation |
| Cost-effectiveness and availability | Price/Availability |
