# Supplementary material for: Current Status and Priorities of Valved Conduits for Right Ventricle-to-Pulmonary Artery Reconstruction in Japan: A Nationwide Survey
Source: Interdiscip Cardiovasc Thorac Surg. 2026 Jun 24;41(7):ivag177. doi: 10.1093/icvts/ivag177 (PMC13324388; doi:10.1093/icvts/ivag177)
Supplement: ivag177_Supplementary_Data [file ivag177_supplementary_data.zip › Supplementary Table 3.docx]

Supplementary Table 3

| Aichi Children's Health and Medical Center |
| --- |
| Chiba Children's Hospital |
| Chiba Municipal Kaihin Hospital |
| Children's Heart Center, Nagoya University Hospital |
| Ehime University Hospital |
| Fukui Cardiovascular Hospital |
| Fukushima Medical University Hospital |
| Gifu County Medical Center |
| Hiroshima City Hiroshima Citizens Hospital |
| Hokkaido University Hospital |
| Hyogo Prefectural Amagasaki General Medical Center |
| Hyogo Prefectural Children's Hospital |
| Ibaraki Children's Hospital |
| Iwate Medical University Hospital |
| JCHO Kyushu Hospital |
| Jichi Medical University Hospital |
| Juntendo University Hospital |
| Kagoshima City Hospital |
| Kanazawa Medical University Hospital |
| Kanazawa University Hospital |
| Keio University Hospital |
| Kitasato University Hospital |
| Kumamoto City Hospital |
| Kurashiki Central Hospital |
| Kurume University Hospital |
| Kyoto Prefectural University of Medicine Hospital |
| Kyushu University Hospital |
| Mie University Hospital |
| Miyagi Children's Hospital |
| National Center for Child Health and Development |
| National Cerebral and Cardiovascular Center |
| Okayama University Hospital |
| Osaka City General Hospital |
| Osaka Medical and Pharmaceutical University Hospital |
| Osaka University Hospital |
| Osaka Women's and Children's Hospital |
| Oyamada Clinic |
| Saiseikai Utsunomiya Hospital |
| Saitama Children's Medical Center |
| Saitama Medical University International Medical Center |
| Shimane University Hospital |
| Shizuoka Children's Hospital |
| Showa University Hospital |
| The University of Tokyo Hospital |
| Toho University Omori Medical Center |
| Tokushima University Hospital |
| Tokyo Metropolitan Children's Medical Center |
| Tokyo Women's Medical University Hospital |
| Uji Tokushukai Medical Center |
| University of Toyama Hospital |
| University of Tsukuba Hospital |
| University of Yamanashi Hospital |
