# Supplementary figures and images for: Current Status and Priorities of Valved Conduits for Right Ventricle-to-Pulmonary Artery Reconstruction in Japan: A Nationwide Survey
Source: Interdiscip Cardiovasc Thorac Surg. 2026 Jun 24;41(7):ivag177. doi: 10.1093/icvts/ivag177 (PMC13324388; doi:10.1093/icvts/ivag177)

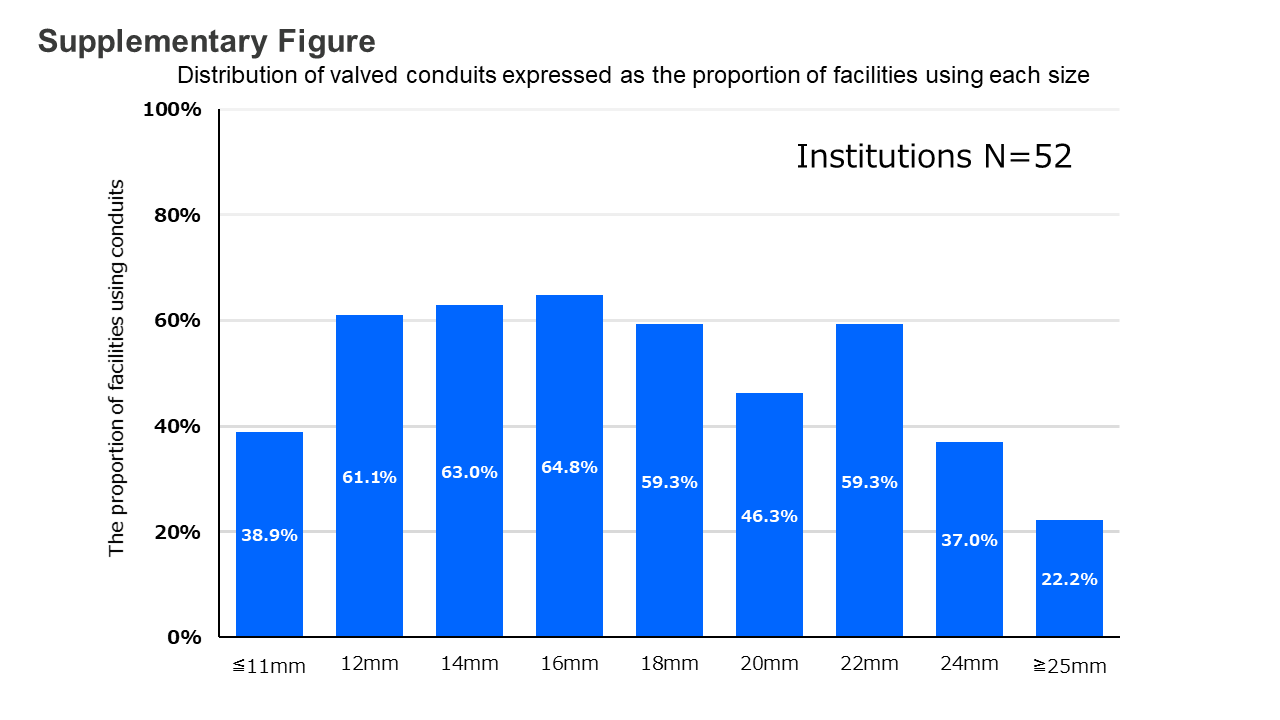

Supplement: ivag177_Supplementary_Data [file ivag177_supplementary_data.zip › Spplementary Figure R1.TIF]
